# Association of Plasma Amyloid, P-Tau, GFAP, and NfL With CSF, Clinical, and Cognitive Features in Patients With Dementia With Lewy Bodies

**DOI:** 10.1212/WNL.0000000000209418

**Published:** 2024-06-03

**Authors:** Katharina Bolsewig, Annemartijn A.J.M. van Unnik, Elena R. Blujdea, Maria C. Gonzalez, Nicholas J. Ashton, Dag Aarsland, Henrik Zetterberg, Alessandro Padovani, Laura Bonanni, Brit Mollenhauer, Sebastian Schade, Rik Vandenberghe, Koen Poesen, Milica G. Kramberger, Claire Paquet, Olivier Bousiges, Benjamin Cretin, Eline A.J. Willemse, Charlotte E. Teunissen, Afina W. Lemstra, for the European-Dementia With Lewy Bodies (E-DLB) Consortium

**Affiliations:** From the Department of Laboratory Medicine (K.B., E.R.B., E.A.J.W., C.E.T.) and Alzheimer Center Amsterdam (A.A.J.M.U., A.W.L.), Amsterdam UMC, the Netherlands; Department of Quality and Health Technology (M.C.G.), University of Stavanger; The Norwegian Centre for Movement Disorders (M.C.G.) and the Centre for Age-Related Medicine (M.C.G., N.J.A., D.A.), Stavanger University Hospital, Norway; Department of Psychiatry and Neurochemistry (N.J.A., H.Z.), the Sahlgrenska Academy at the University of Gothenburg, Mölndal, Sweden; Department of Old Age Psychiatry (N.J.A., D.A.), King's College London, United Kingdom; Clinical Neurochemistry Laboratory (H.Z.), Sahlgrenska University Hospital, Mölndal, Sweden; Department of Neurodegenerative Disease (H.Z.), UCL Institute of Neurology; UK Dementia Research Institute at UCL (H.Z.), London, United Kingdom; Hong Kong Center for Neurodegenerative Diseases (H.Z.), Hong Kong, China; Wisconsin Alzheimer's Disease Research Center (H.Z.), University of Wisconsin School of Medicine and Public Health, Madison; Neurology Unit (A.P.), Department of Clinical and Experimental Sciences, University of Brescia, Italy; Department of Medicine and Aging Sciences (L.B.), University G. d'Annunzio of Chieti-Pescara, Chieti, Italy; Department of Neurology (B.M.), University Medical Center Göttingen; Paracelsus-Elena-Klinik (B.M., S.S.), Germany; Department of Neurosciences (R.V., K.P.), KU Leuven, Belgium; Department of Neurology and Medical Faculty (M.G.K.), University Medical Center Ljubljana, Slovenia; Department of Neurobiology (M.G.K.), Karolinska Institutet, Huddinge, Sweden; Université de Paris Cité (C.P.), Centre de Neurologie Cognitive, Paris; Laboratory of Biochemistry and Molecular Biology (O.B.), University Hospital of Strasbourg; University of Strasbourg and CNRS (O.B., B.C.); Memory Resource and Research Centre (B.C.), University Hospital of Strasbourg, France; Department of Neurology (E.A.J.W.), Multiple Sclerosis Center; Research Center for Clinical Neuroimmunology and Neuroscience Basel (E.A.J.W.); and Departments of Biomedicine and Clinical Research (E.A.J.W.), University Hospital Basel and University of Basel, Switzerland.

## Abstract

**Background and Objectives:**

Plasma β-amyloid-1–42/1–40 (Aβ42/40), phosphorylated-tau (P-tau), glial fibrillary acidic protein (GFAP), and neurofilament light (NfL) have been widely examined in Alzheimer disease (AD), but little is known about their reflection of copathologies, clinical importance, and predictive value in dementia with Lewy bodies (DLB). We aimed to evaluate associations of these biomarkers with CSF amyloid, cognition, and core features in DLB.

**Methods:**

This cross-sectional multicenter cohort study with prospective component included individuals with DLB, AD, and healthy controls (HCs), recruited from 2002 to 2020 with an annual follow-up of up to 5 years, from the European-Dementia With Lewy Bodies consortium. Plasma biomarkers were measured by single-molecule array (Neurology 4-Plex E kit). Amyloid status was determined by CSF Aβ42 concentrations, and cognition was assessed by Mini-Mental State Examination (MMSE). Biomarker differences across groups, associations with amyloid status, and clinical core features were assessed by analysis of covariance. Associations with cognitive impairment and decline were assessed by linear regression and linear mixed-effects models.

**Results:**

In our cohort consisting of 562 individuals (HC n = 89, DLB n = 342, AD n = 131; 250 women [44.5%], mean [SD] age of 71 [8] years), sex distribution did not differ between groups. Patients with DLB were significantly older, and had less years of education and worse baseline cognition than HC, but not AD. DLB participants stratified for amyloid status differed significantly in plasma Aβ42/40 ratio (decreased in amyloid abnormal: β = −0.008, 95% CI −0.016 to −0.0003, *p* = 0.01) and P-tau (increased in amyloid abnormal, P-tau181: β = 0.246, 95% CI 0.011–0.481; P-tau231: β = 0.227, 95% CI 0.035–0.419, both *p* < 0.05), but not in GFAP (β = 0.068, 95% CI −0.018 to 0.153, *p* = 0.119), and NfL (β = 0.004, 95% CI −0.087 to 0.096, *p* = 0.923) concentrations. Higher baseline GFAP, NfL, and P-tau concentrations were associated with lower MMSE scores in DLB, and GFAP and NfL were associated with a faster cognitive decline (GFAP: annual change of −2.11 MMSE points, 95% CI −2.88 to −1.35 MMSE points, *p* < 0.001; NfL: annual change of −2.13 MMSE points, 95% CI −2.97 to −1.29 MMSE points, *p* < 0.001). DLB participants with parkinsonism had higher concentrations of NfL (β = 0.08, 95% CI 0.02–0.14, *p* = 0.006) than those without.

**Discussion:**

Our study suggests a possible utility of plasma Aβ42/40, P-tau181, and P-tau231 as a noninvasive biomarkers to assess amyloid copathology in DLB, and plasma GFAP and NfL as monitoring biomarkers for cognitive symptoms in DLB.

## Introduction

Dementia with Lewy bodies (DLB) is considered the most frequent type of dementia after Alzheimer disease (AD) in the elderly.^1^ It is clinically characterized by cognitive decline and visual hallucinations, cognitive fluctuations, sleep disturbances, parkinsonism, and autonomic dysfunction.^2^ Survival after diagnosis has shown to be shorter for patients with DLB when compared with AD. In DLB, survival times of about 4 years after diagnosis have been reported; however, large heterogeneity of clinical presentation and progression, and copathologies can hamper prediction of the disease course over time.^3-6^

The neuropathologic hallmark of DLB is the accumulation of phosphorylated α-synuclein (α-syn) aggregates in intracytoplasmic inclusions, so-called Lewy bodies and Lewy neurites, throughout the brain.^1^ In addition, concomitant AD neuropathology is observed in a substantial portion of patients with DLB,^7^ and there is considerable overlap in both clinical and pathologic features between DLB and AD and Parkinson disease (PD). Furthermore, pathologic processes other than protein aggregation are present in DLB and probably contribute to neurodegeneration and disease progression. For instance, accumulating evidence suggests that α-syn, but also AD-pathology, evokes neuroinflammation in DLB.^8^ Therefore, there is a need for combined biomarker assessment reflecting these different disease processes to define the effect of these processes on diagnosis, heterogeneity in disease manifestation, and disease progression.

CSF analyses and PET imaging are considered state of the art for detecting AD pathology, but recent developments have led to plasma biomarkers for the detection of several cerebral pathologic processes with adequate accuracy. With current multiplex techniques, several biomarkers can be measured simultaneously in a single sample, greatly improving the possibilities to investigate the effect of different pathologies in a less invasive and time-consuming fashion.^9^

In numerous cohorts, blood-based core AD biomarkers correlated highly with CSF AD biomarkers in all stages of AD.^10^ In addition to core AD biomarkers (decreased β-amyloid [Aβ] 42/40 ratio and increased phosphorylated-tau [P-tau] concentrations), other more general biomarkers including neurofilament light (NfL) as a marker for neuroaxonal damage and glial fibrillary acidic protein (GFAP) reflecting reactive astrogliosis are elevated in both patients with DLB and AD compared with HC.^11-13^

Most studies analyze blood-based biomarkers with a main focus on AD, and therefore, less is known about the use of these biomarkers as early diagnostic and prognostic markers in DLB. Although some studies include a DLB group to evaluate the diagnostic performance of blood-based biomarkers for the differentiation of AD from DLB, sample sizes are rather moderate and studies of blood-based biomarkers in DLB specifically are scarce.^14-16^

The differential power of blood biomarkers to differentiate AD from DLB seems to be rather cohort-dependent.^14^ Although, in a previous study P-tau181 and GFAP have been found to be associated with CSF amyloid status, another study reported no association of any of the above-mentioned biomarkers with amyloid PET. Notably, both studies included rather small sample sizes (association with CSF amyloid: n = 31 amyloid normal (A−) vs n = 18 amyloid abnormal (A+); association with amyloid PET: n = 30 A− vs n = 29 A+).^15,16^

Recently, we demonstrated in a large European cohort that plasma concentrations of specific P-tau species (P-tau181 and P-tau231) are significantly higher in patients with DLB compared with healthy controls (HCs), but lower compared with AD.^17^ Furthermore, we found that plasma P-tau biomarkers are elevated in DLB participants with concordant amyloid copathology and are associated with cognitive decline in the whole DLB group. These results suggest that P-tau species are reflective of brain amyloid because it has been shown before in various AD cohorts^18,19^ and could play an important role in monitoring disease progression in patients with DLB. The next question is whether blood biomarkers reflecting amyloidosis and other neurodegenerative processes including neuroaxonal damage and reactive astrogliosis are associated with AD copathology, cognition, or core features in DLB.

In this study, we aimed to add to and put our previous findings on P-tau into context, by evaluating the association of the plasma biomarkers, Aβ42/40 ratio, NfL, and GFAP, all reflecting different disease processes, with AD copathology, clinical features, and progression of cognition in DLB, leveraging a large multicenter cohort from the European-Dementia With Lewy Bodies (E-DLB) consortium.

## Methods

### Study Population

**Figure 1:**
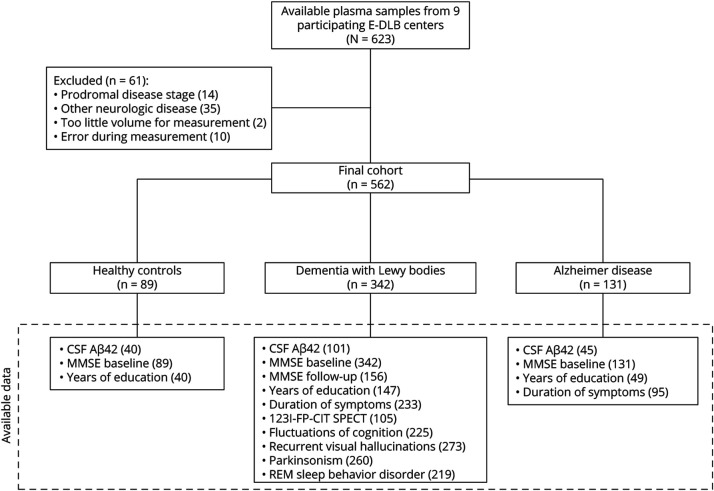
Overview of Sample Availability, Cohort Selection, and Data Availability Per Diagnostic Group The diagram depicts number of available samples, exclusion criteria, and number of participants in the final cohort, as well as available data for each diagnostic group.

We retrospectively included participants from the E-DLB initiative cohort, from 9 different participating centers (eTable 1). Participants were referred between 2002 and 2020 from outpatient clinics for memory, movement disorders, geriatric medicine, psychiatry, or neurology as part of their diagnostic work-up.^17,20^ In total, plasma samples of 623 individuals were available from the E-DLB consortium. We excluded patients with other neurologic diseases, in prodromal disease stages, samples with too little volume, and those samples that gave an error during measurement. This resulted in a cohort of 562 individuals including 342 patients with probable DLB, 131 patients with AD, and 89 HC. Diagnoses of AD and probable DLB were harmonized between centers and made according to consensus guidelines^2,21^ based on all available clinical and diagnostic test results, by a multidisciplinary team, a group of at least 2 clinical experts, or by the treating physician. In 83 (79.0%) of 105 individuals with available 123I-N-ω-fluoropropyl-2β-carbomethoxy-3β-(4-iodophenyl) nortropane (123I-FP-CIT) SPECT scans, the clinical diagnosis of DLB was supported by abnormal dopamine transporter activity. Plasma biomarkers studied in this study were not used to support diagnoses. HC were individuals who presented at the respective clinic but did not have any neurologic disease. An overview of sample availability, cohort selection criteria, and data availability per diagnostic group is presented in Figure 1.

Participating centers and sample contribution are listed in eTable 1. Cognitive impairment at baseline was assessed by the Mini-Mental State Examination (MMSE),^22^ and follow-up MMSE acquisition was performed in a subgroup of the patients with DLB at least once, and up to 5 years after the initial visit (n = 156 [5 years: n = 44, 4 years: n = 24, 3 years: n = 4, 2 years: n = 35, 1 year: n = 29], eFigure 1). Furthermore, in subsets of the DLB group, DLB core feature data were assessed at baseline as described previously^23-26^ and subsequently dichotomized as “present” or “not present” (fluctuations of cognition: n = 225 [present in n = 171, 76.0%], recurrent visual hallucinations: n = 273 [present in n = 149, 54.6%], parkinsonism: n = 260 [present in n = 161, 61.9%], and REM sleep behavior disorder [RBD]: n = 219 [present in n = 125, 57.1%]). Amyloid copathology was assessed in 101 of 342 patients with DLB (29.5% of the total DLB group) of whom 48 (47.5%) were defined abnormal (A+) by decreased CSF Aβ42 concentrations and 53 (52.5%) had normal CSF Aβ42 concentrations (A−). In the AD group, CSF Aβ42 concentrations were measured in 45 patients (34.4% of the total AD group) and CSF findings were in concordance with the clinical diagnosis for all patients with AD. All participants in the HC group with CSF amyloid data available (n = 40) were A−. Cutoff values and assays used for CSF Aβ42 measurements and baseline cohort characteristics for those individuals with CSF Aβ42 assessment are listed in eTables 2 and 3. Information on the duration of experienced symptoms was available for 233 patients with DLB and 95 patients with AD, while information on years of education was available for 147 patients with DLB, 49 patients with AD, and 40 HC.

### Plasma Biomarkers

Information about plasma collection and sample handling is reported in eTable 2. Concentrations of Aβ40, Aβ42, GFAP, and NfL in plasma were measured on the Simoa HD-X Analyzer (Quanterix, Billerica, MA), with the commercially available Neurology 4-Plex E kit (Quanterix) at the Neurochemistry Laboratory, Amsterdam University Medical Centers, the Netherlands. The operating staff was blinded to the clinical data. Before measurement, samples were centrifuged at 10,000*g* for 10 minutes. Samples were measured across 3 runs in singlicate. Repeatability and intermediate precision were determined based on 3 quality controls and fell within the accepted range CV% <15%, for all markers (mean CV% [SD] repeatability, Aβ40: 2.7% [0.7%], Aβ42: 4.2% [1.4%], GFAP 8.2% [4.3%], NfL 5.6% [0.9%]; mean CV% [SD] intermediate precision, Aβ40: 3.3% [0.7%], Aβ42: 6.7% [0.5%], GFAP: 9.7% [3.6%], NfL: 6.7% [0.5%]). Plasma P-tau181 and P-tau231 were measured as described previously.^17^

### Statistical Analysis

All statistical analyses were conducted with R version 4.0.3.^27^ All tests were 2-tailed, corrected for multiple testing by Bonferroni correction, and the significance level was set to α = 0.05. Distributions of variables and model residuals were assessed by visual examination and the Shapiro-Wilk test (pastecs R package^28^). To achieve a normal distribution, P-tau 181, P-tau231, GFAP, and NfL were log10-transformed. Biomarker differences across diagnostic groups were assessed with analysis of covariance (car package), corrected for age and sex, and subsequent Tuckey post hoc test (multcomp R package^29^). Sex-related biomarker differences were assessed by the *t* test. Similarly, associations of plasma biomarkers with CSF amyloid status and DLB core features were assessed in the respective DLB subsets. Associations of plasma biomarkers with cognitive impairment at baseline were determined by linear regression analyses. Longitudinal cognitive decline was analyzed in patients with DLB with at least 1 follow-up measurement of MMSE scores, with linear mixed-effects models (lmerTest R package^30^) with random intercept and slope for each individual. First, we assessed the association of years of education with cognitive decline independently of plasma biomarkers values, by adding an interaction term of years of education and follow-up time. Subsequently, the association of plasma biomarker levels with cognitive decline was assessed by adding an interaction of plasma biomarkers and follow-up time, correcting the models for age, sex, and years of education. Model performances were assessed using the Akaike information criterion (AIC) and analysis of variance.

### Standard Protocol Approvals, Registrations, and Patient Consents

All participants provided written informed consent for their medical data and biomaterials to be used for research purposes, and the study was approved by the ethical committee of each participating center (eTable 1).

### Data Availability

Data access to anonymized patient-level data can be requested from the corresponding author on reasonable request.

## Results

### Demographics

Baseline characteristics and plasma biomarker levels are summarized in Table 1. No difference in sex distribution was observed across diagnostic groups. The DLB and AD group did not differ significantly in age, years of education, duration of symptoms, or baseline MMSE scores, and no significant difference in years of education was observed between AD and HC. Patients with DLB and AD were significantly older than HC (mean age [SD]; HC: 67.4 [7.3], DLB: 71.6 [8.1], AD: 71.9 [7.9]) and had lower baseline MMSE scores. Participants with DLB had significantly less years of education compared with HC.

**Table 1:** Baseline Cohort Characteristics

Baseline characteristics	DLB (n = 342 [60.9%])	AD (n = 131 [23.3%])	HC (n = 89 [15.8%])	*p* Value
Sex, n (%)				
Female	144 (42.1)	65 (49.6)	41 (46.1)	0.33
Male	198 (57.9)	66 (50.4)	48 (53.9)	
Age, y, mean (SD)	71.6 (8.1)	71.9 (7.9)	67.4 (7.3)	<0.001^a,b^
Years of education, y, mean (SD)^c^	10.1 (4.3)	11.2 (3.5)	12.6 (3.3)	0.001^a^
Duration of symptoms, mo, median (IQR)^d^	12.0 (3.0–36.0)	7.0 (2.0–36.0)	NA	0.11
MMSE score, median (IQR)	23.0 (18.4–26.3)	23.0 (19.0–26.0)	29.0 (27.0–30.0)	<0.001^a,b^
Abnormal CSF Aβ42 status, n (%)^e^	48 (47.5)	45 (100.0)	0 (0.0)	NA
Abnormal 123I-FP-CIT SPECT scan, n (%)^f^	83 (79.0)	NA	NA	NA
Plasma biomarkers				
Aβ42/Aβ40 ratio, mean (SD)	0.063 (0.015)	0.056 (0.012)	0.065 (0.014)	<0.001^b,g^
GFAP, pg/mL, median (IQR)	173 (108–255)	183 (133–246)	99.3 (71.3–140)	<0.001^a,b^
NfL, pg/mL, median (IQR)	27.8 (19.4–39.6)	26.3 (19.4–35.1)	16.1 (12.4–24.3)	<0.001^a,b^
P-tau181, pg/mL, median (IQR)	15.9 (11.8–22.5)	21.1 (16.1–26.5)	10.6 (8.8–13.1)	<0.001^a,b,g^
P-tau231, pg/mL, median (IQR)	11.4 (8.4–15.7)	15.6 (11.0–20.0)	7.6 (5.8–9.4)	<0.001^a,b,g^
Presence of DLB core features, n (%)				
Fluctuations of cognition (total n = 225)	171 (76.0)	NA	NA	NA
Recurrent visual hallucinations (total n = 273)	149 (54.6)	NA	NA	NA
Parkinsonism (total n = 260)	161 (61.9)	NA	NA	NA
REM sleep behavior disorder (total n = 219)	125 (57.1)	NA	NA	NA

Abbreviations: 123I-FP-CIT = 123I-N-ω-fluoropropyl-2β-carbomethoxy-3β-(4-iodophenyl) nortropane; Aβ = β-amyloid; AD = Alzheimer disease; DLB = dementia with Lewy bodies; GFAP = glial fibrillary acidic protein; HC = healthy control; MMSE = Mini-Mental State Examination; NA = not available; NfL = neurofilament light; P-tau = phosphorylated tau.

a Differences between DLB and HC.

b Differences between AD and HC.

c Years of education were assessed in 147 patients with DLB, 49 patients with AD, and 40 HC.

d Duration from first cognitive or motor symptoms was assessed in 233 patients with DLB, and 95 patients with AD.

e CSF Aβ42 status was assessed in 101 patients with DLB, 45 patients with AD, and 40 HC.

f 123I-FP-CIT SPECT scan was performed in 105 patients with DLB.

g Differences between DLB and AD.

### Plasma Biomarkers Across Diagnostic Groups

**Figure 2:**
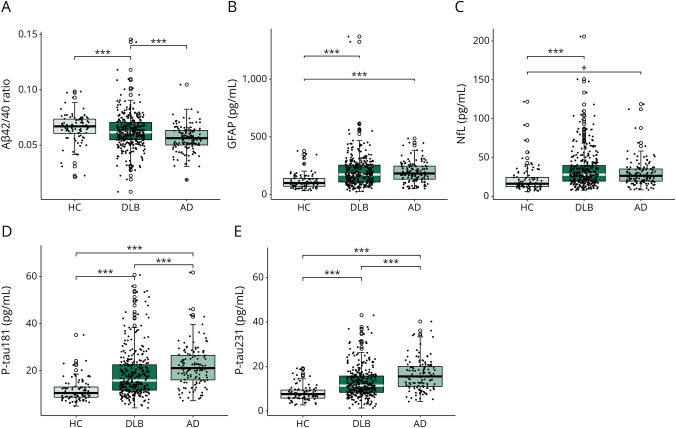
Plasma Biomarkers Across Diagnostic Groups Plasma biomarker differences of Aβ42/Aβ40 ratio (A), GFAP (B), NfL (C), P-tau181 (D), and P-tau231 (E) across diagnostic groups. Plasma Aβ42/Aβ40 ratio was significantly higher in patients with DLB and HCs compared with patients with AD. Both plasma P-tau species were significantly higher in DLB compared with HC and significantly lower in DLB and HC compared with AD. Plasma GFAP and NfL concentrations were significantly higher in DLB and AD compared with HC. Plasma biomarker differences were assessed across groups by ANCOVA corrected for age and sex, and subsequent post hoc analysis by the Tukey post hoc test. *p* Values were adjusted for multiple comparison. *p < 0.05, ***p < 0.001. Aβ = β-amyloid; AD = Alzheimer disease; ANCOVA = analysis of covariance; DLB = dementia with Lewy bodies; GFAP = glial fibrillary acidic protein; HC = healthy control; MMSE = Mini-Mental State Examination; NfL = neurofilament light; P-tau = phosphorylated tau.

Biomarker differences across diagnostic groups are visualized in Figure 2. The Aβ42/40 ratio was significantly higher in the overall DLB group (β = 0.007, 95% CI 0.003–0.01, *p* < 0.001) and HC (β = 0.009, 95% CI 0.004–0.014, *p* < 0.001) compared with AD. GFAP and NfL concentrations were significantly higher in DLB (GFAP: β = 0.165, 95% CI 0.105–0.225, *p* < 0.001; NfL: β = 0.130, 95% CI 0.070–0.190, *p* < 0.001) and AD (GFAP: β = 0.168, 95% CI 0.099–0.237, *p* < 0.001; NfL: β = 0.086, 95% CI 0.016–0.155, *p* < 0.05) compared with HC. Both P-tau species were significantly increased in DLB (P-tau181: β = 0.368, 95% CI 0.245–0.491, *p* < 0.001; P-tau231: β = 0.379, 95% CI 0.242–0.517, *p* < 0.001) and AD (P-tau181: β = 0.579, 95% CI 0.437–0.720, *p* < 0.001; P-tau231: β = 0.635, 95% CI 0.476–0.793, *p* < 0.001) compared with HC, and higher in AD compared with DLB (P-tau181: β = 0.211, 95% CI 0.106–0.315, *p* < 0.001; P-tau231: β = 0.2552, 95% CI 0.138–0.372, *p* < 0.001). In the total cohort and within the AD group, GFAP was significantly higher in women than men (total cohort: *t*_500.38_ = −3.62, *p* = 0.001; AD: *t*_127.33_ = −3.64, *p* = 0.002). No other sex-related biomarker differences were observed.

### Association of Plasma Biomarkers With CSF Amyloid Status

**Figure 3:**
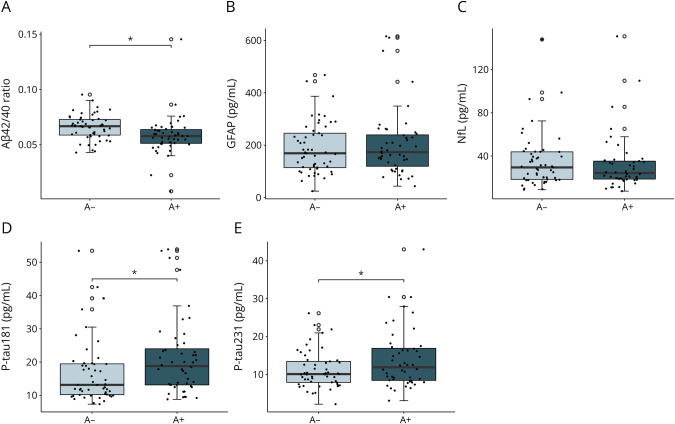
Plasma Biomarkers in CSF Aβ42 Abnormal DLB Participants Plasma biomarkers Aβ42/40 ratio (A), GFAP (B), NfL (C), P-tau181 (D), and P-tau231 (E) in patients with DLB by CSF Aβ42 status (A+: n = 48; A−: n = 53). Plasma Aβ42/40 ratio was significantly lower, and both plasma P-tau species were significantly higher in DLB A+ patients compared with DLB A− patients. Plasma GFAP and NfL concentrations did not differ between A− and A+ patients with DLB. Plasma biomarkers were compared between groups by ANCOVA corrected for age and sex. *p < 0.05. Aβ = β-amyloid; AD = Alzheimer disease; ANCOVA = analysis of covariance; DLB = dementia with Lewy bodies; GFAP = glial fibrillary acidic protein; HC = healthy control; MMSE = Mini-Mental State Examination; NfL = neurofilament light; P-tau = phosphorylated tau.

DLB A+ patients had significantly lower plasma Aβ42/40 ratio, and higher P-tau181 and P-tau231 levels than DLB A− patients (Aβ42/40: β = −0.008, 95% CI −0.016 to −0.0003, *p* = 0.01; P-tau181: β = 0.246, 95% CI 0.011–0.481, *p* < 0.05; P-tau231: β = 0.227, 95% CI 0.035–0.419, *p* < 0.05), but no significant difference could be observed for GFAP (β = 0.068, 95% CI −0.018 to 0.153, *p* = 0.119) and NfL (β = 0.004, 95% CI −0.087 to 0.096, *p* = 0.923) (Figure 3). In DLB A+ individuals, the Aβ42/40 ratio was significantly lower (β = −0.007, 95% CI −0.014 to −0.0001, *p* = 0.045) compared with HC, but not significantly different from AD (β = 0.002, 95% CI −0.005 to 0.008, *p* = 0.937), and in DLB A− individuals, the Aβ42/40 ratio was significantly higher (β = 0.010, 95% CI 0.004–0.016, *p* < 0.001) compared with AD, but not significantly different from HC (β = 0.001, 95% CI −0.006 to 0.008, *p* = 0.995). The P-tau species were significantly increased in DLB A− (P-tau181: β = 0.272, 95% CI 0.065–0.479, *p* < 0.01; P-tau231: β = 0.274, 95% CI 0.044–0.504, *p* < 0.05) and DLB A+ (P-tau181: β = 0.518, 95% CI 0.308–0.728, *p* < 0.001; P-tau231: β = 0.478, 95% CI 0.242–0.714, *p* < 0.001) compared with HC, and significantly lower in DLB A− compared with AD (P-tau181: β = −0.304, 95% CI −0.496 to −0.111, *p* < 0.001; P-tau231: β = −0.359, 95% CI −0.573 to −0.145, *p* < 0.001).

### Association of Plasma Biomarkers With Cognitive Impairment and Decline

In the DLB group, GFAP, NfL, P-tau181, and P-tau231, but not Aβ42/40 ratio, were associated with global cognitive impairment (MMSE) at baseline (Aβ42/40: β = −13.18, 95% CI −56.42 to 30.05, *p* = 0.549; GFAP: β = −9.25, 95% CI −12.06 to −6.44, *p* < 0.001; NfL: β = −7.41, 95% CI −10.28 to −4.54, *p* < 0.001; P-tau: β = −2.61, 95% CI −3.99 to −1.24, *p* < 0.001; P-tau: β = −2.42, 95% CI −3.67 to −1.17, *p* < 0.001) when corrected for age and sex. On additional correction for years of education, this effect remained significant for GFAP (β = −4.11, 95% CI −8.06 to −0.16, *p* = 0.04) and P-tau181 (β = −1.75, 95% CI −3.49 to −0.01, *p* < 0.05), but lost significance for NfL (β = −2.58, 95% CI −6.30 to −1.14, *p* = 0.17) and P-tau231 (β = −1.48, 95% CI −3.19 to 0.23, *p* = 0.09). Similar associations were found in the AD group, but no association with cognition at baseline was found in HC (Table 2).

**Table 2:** Associations of Baseline Plasma Biomarkers With Global Cognition at Baseline After Correction for Age and Sex

	HC		DLB		AD	
	Estimate (95% CI)	*p* Value	Estimate (95% CI)	*p* Value	Estimate (95% CI)	*p* Value
Aβ42/40	−9.64 (−41.59 to 22.31)	0.549	−13.18 (−56.42 to 30.05)	0.549	−1.64 (−88.87 to 85.59)	0.970
GFAP^a^	0.93 (−1.37 to 3.23)	0.424	−9.25 (−12.06 to −6.44)	<0.001	−11.54 (−16.20 to −6.87)	<0.001
NfL^a^	0.55 (−1.58 to 2.68)	0.610	−7.41 (−10.28 to −4.54)	<0.001	−10.88 (−15.92 to −5.84)	<0.001
P-tau181^a^	0.56 (−0.86 to 1.98)	0.437	−2.61 (−3.99 to −1.24)	<0.001	−4.25 (−6.79 to −1.70)	<0.01
P-tau231^a^	−0.05 (−1.14 to 1.04)	0.922	−2.42 (−3.67 to −1.17)	<0.001	−4.92 (−7.08 to −2.76)	<0.001

Abbreviations: Aβ = β-amyloid; AD = Alzheimer disease; DLB = dementia with Lewy bodies; GFAP = glial fibrillary acidic protein; HC = healthy control; NfL = neurofilament light; P-tau = phosphorylated tau.

a Biomarker concentrations were log-transformed.

**Figure 4:**
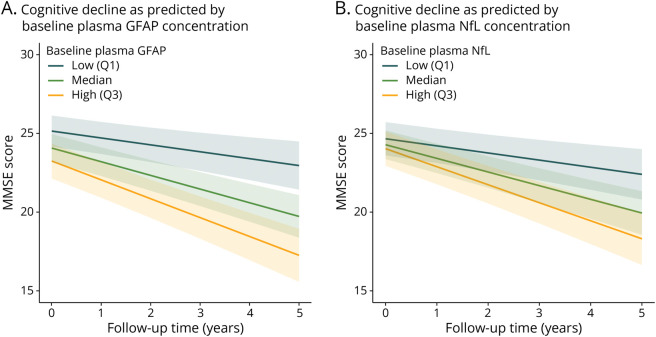
Associations of Baseline Plasma GFAP and NfL With Cognitive Decline in Patients With Dementia With Lewy Bodies Association of cognitive decline with baseline plasma GFAP (A) and NfL (B) concentrations. Linear mixed-effects models, corrected for the effect of age and sex, were built to analyze the association of baseline plasma biomarker concentrations with cognitive decline, as measured by MMSE scores over time, for up to 5 years. The line represents the estimated marginal model of decrease in MMSE scores over up to 5 years of follow-up time with different baseline plasma biomarker concentrations. To illustrate the association of different baseline biomarker concentrations with rate of cognitive decline, low and high biomarker concentrations were defined as the first (Q1) and third quartile (Q3), respectively. The transparent areas show 95% CIs around the mean estimated values. AD = Alzheimer disease; GFAP = glial fibrillary acidic protein; HC = healthy control; MMSE = Mini-Mental State Examination; NfL = neurofilament light.

Longitudinal MMSE data were available for 156 DLB individuals with follow-up times of up to 5 years (5 years: n = 44, 4 years: n = 24, 3 years: n = 24, 2 years: n = 35, 1 year: n = 29), with a mean (SD) follow-up time of 3.1 (1.5) years. Years of education were not associated with the rate of cognitive decline (β = −0.03, 95% CI −0.14 to 0.08, *p* = 0.57; eFigure 2). Although Aβ42/40 was not associated with cognitive decline in the DLB group, GFAP concentration was associated with an annual change of −2.11 MMSE points (95% CI −2.88 to −1.35 MMSE points, *p* < 0.001) and NfL concentration was associated with an annual change of −2.13 MMSE points (95% CI −2.97 to −1.29 MMSE points, *p* < 0.001) (Figure 4) after correction for age and sex. After additional correction for years of education, these associations lost significance (GFAP: β = −0.40, 95% CI −3.12 to 2.34, *p* = 0.78; NfL: β = −1.92, 95% CI −4.14 to 0.38, *p* = 0.10) and the addition of NfL to the GFAP model did not improve the model performance (AIC 3,116.4 vs 3,116.1).

### Association of Plasma Biomarkers With DLB Core Features and 123I-FP-CIT SPECT Status

DLB core features are summarized in Table 1. None of the biomarkers were associated with the presence of visual hallucinations or RBD in the DLB group. Higher concentration of NfL was associated with the presence of parkinsonism (β = 0.08, 95% CI 0.02–0.14, *p* = 0.006), and lower P-tau231 was associated with fluctuations of cognition (β = −0.18, 95% CI −0.33 to −0.02, *p* < 0.05; eFigure 3). These associations were not significant for any of the other plasma biomarkers. Levels of plasma biomarkers did not differ between 123I-FP-CIT SPECT status normal and abnormal individuals (eFigure 4). Graphs showing each plasma biomarker in association with DLB core features and 123I-FP-CIT SPECT status are presented in eFigure 3.

## Discussion

In this study, we evaluated the association of plasma Aβ42/40, GFAP, and NfL with amyloid copathology, progression of cognition, and clinical core features of DLB in a large E-DLB multicenter cohort. We found that the plasma Aβ42/40 ratio was higher in patients with DLB compared with AD and not significantly different from HC, while plasma GFAP and NfL were higher in patients with DLB and AD compared with HC, but not significantly different between DLB and AD, and P-tau181 and P-tau231 were significantly higher in DLB compared with HC and significantly lower in DLB and HC compared with AD. In DLB, higher GFAP and NfL at baseline were associated with cognitive impairment and predicted faster cognitive decline. Only the Aβ42/40 ratio and both P-tau species were associated with CSF Aβ42 status in DLB. Increased plasma NfL concentrations were associated with the presence of parkinsonism, and increased P-tau231 concentrations were present in the absence of fluctuations of cognition.

NfL is released into body fluids on axonal damage, indicating neurodegenerative processes.^31^ GFAP is known to be a marker for astrocyte activation, a pathologic process involved in various neurologic diseases and specifically associated with amyloid pathology in AD.^32^ Increased concentrations of blood NfL and GFAP have previously been shown in both AD^33-35^ and DLB (total study group n = 300, of which n = 110 DLB).^16^ The increase of plasma GFAP in DLB, without being associated with amyloid copathology in the DLB group, could indicate that GFAP may also reflect different processes in DLB compared with AD, such as neurodegeneration and/or α-syn pathology.

The increase in plasma NfL and GFAP concentrations was associated with cognitive impairment and a more rapid cognitive decline in the DLB group, with a stronger association for GFAP. In AD, this relationship has been shown before.^34,36^ On correction for years of education, this association lost significance, which is most likely due to a loss of power caused by missing data (n = 98 (62.8%) of n = 156). As described by the cognitive reserve theory, higher educational levels are believed to extend the time to cognitive decline.^37^ However, it has been proposed before that the rate of neurodegenerative processes is not influenced by educational levels.^38^ Instead, it is possible that although patients of higher education would have higher baseline MMSE levels, the rate of cognitive decline would still be comparable with that of patients with lower education and similar baseline plasma NfL or GFAP concentrations. In our data, we could observe that indeed the rate of cognitive decline was not altered on stratification for educational levels (eFigure 2). These findings could indicate that NfL and particularly GFAP as markers for neurodegeneration and astrogliosis, respectively, could have a value for monitoring cognition in DLB. Although higher levels of both P-tau species were associated with worse cognitive impairment at baseline, associations with cognitive decline did not reach significance, unlike in a previous report.^17^ This loss of significance is likely explained by the inclusion of only a subgroup of the initial E-DLB cohort in this study.

As expected, the plasma Aβ42/40 ratio was decreased, and both P-tau species were increased in amyloid positive DLB individuals, as defined by CSF Aβ42, indicating plasma Aβ42/40 ratio, P-tau181, and P-tau231 to be suitable, less invasive markers to assess amyloid copathology. This association was not observed for plasma GFAP. Already in early stages of AD, a close relationship between GFAP concentration and amyloid burden, as determined by Aβ PET or CSF Aβ42/40 ratio, has been shown in several studies.^33,35,39^ In DLB, however, inconsistent findings concerning the association of GFAP with Aβ PET have been reported.^16,40^ As previously suggested, other pathologic processes, such as α-syn deposition causing neuroinflammation,^8^ could affect astrogliosis,^41-44^ possibly masking the association of GFAP concentration with amyloid burden in DLB.

These findings suggest the utility of plasma Aβ42/40, P-tau181, and P-tau231 to assess amyloid abnormality, and NfL and GFAP to monitor disease progression in DLB. Although the general trend of biomarker levels across groups is coherent with previous studies,^14-16^ we still observed large heterogeneity of biomarker concentrations within, and substantial overlap of biomarker concentrations between groups, which diminishes their utility for differential diagnosis of DLB.

We show that patients with DLB with parkinsonism present with higher plasma NfL concentration than those without parkinsonism. These results corroborate previous results of increased plasma NfL concentration in atypical parkinsonism disorders^45^ and predicting faster motor progression in PD,^46^ possibly caused by more pronounced neurodegeneration. The lack of such associations with other core features, such as recurrent visual hallucinations and RBD, could suggest that these features may have a stronger functional component.^47-49^

Strengths of this study include the large sample size, the longitudinal data for a subgroup, and multicenter design. Our study also faced some limitations. Owing to the retrospective and naturalistic character of our study, some variables included missing data, only allowing for certain analyses in subsets of the cohort, for example, CSF amyloid measurements were based on Aβ42 alone and available in 45 patients with AD and 101 patients with DLB. Of the included patients with DLB with available 123I-FP-CIT SPECT scan information, 21.0% (n = 22) were rated as normal. It has been shown that normal 123I-FP-CIT SPECT scans are found in around 10% of patients with DLB and can convert to abnormal over time.^50^ Furthermore, individuals with normal 123I-FP-CIT SPECT did not show significantly different biomarker concentrations compared with 123I-FP-CIT SPECT abnormal patients with DLB (data not shown), but the possible inclusion of misdiagnosed cases cannot be ruled out. Furthermore, we evaluated association of biomarkers with cognitive decline based on MMSE. This measure is rather domain-unspecific and more specific assessments could give more detailed insights into disease progression. In addition, although all statistical analyses were corrected for the confounding effect of age, it is important to emphasize that this might not fully eliminate the effect of the control group being of significant younger age compared with both disease groups.

Our findings suggest the possible utility of plasma GFAP and NfL for monitoring disease progression in patients with DLB in the future, which would make them relevant outcomes in clinical trials. Further longitudinal studies and analyzing the association with other disease progression measures such as survival rate will be needed to verify our findings. Adding to the previous findings for P-tau181 and P-tau231,^17^ we found an association of plasma Aβ42/40 ratio and amyloid copathology in DLB, indicating a possible utility of these markers to assess amyloid copathology in DLB as a less invasive alternative to CSF. These markers could become beneficial for patient selection and stratification in clinical trials, patient selection for anti-amyloid treatment, and could aid in understanding the influence of amyloid copathology on disease progression and survival in DLB. However, validation of our findings in amyloid PET abnormal DLB participants would be valuable, further correlation studies in DLB are needed, and cutoff values need to be established.

## Appendix 1 Authors

**Table:**

Name	Location	Contribution
Katharina Bolsewig, MSc	Department of Laboratory Medicine, Amsterdam UMC, the Netherlands	Drafting/revision of the manuscript for content, including medical writing for content; study concept or design; analysis or interpretation of data
Annemartijn A.J.M. van Unnik, MD	Alzheimer Center Amsterdam, Amsterdam UMC, the Netherlands	Drafting/revision of the manuscript for content, including medical writing for content
Elena R. Blujdea, MSc	Department of Laboratory Medicine, Amsterdam UMC, the Netherlands	Drafting/revision of the manuscript for content, including medical writing for content; major role in the acquisition of data
Maria C. Gonzalez, MD	Department of Quality and Health Technology, University of Stavanger; The Norwegian Centre for Movement Disorders, Stavanger University Hospital; Centre for Age-Related Medicine, Stavanger University Hospital, Norway	Drafting/revision of the manuscript for content, including medical writing for content; major role in the acquisition of data; additional contributions: administrative, technical, or material support
Nicholas J. Ashton	Centre for Age-Related Medicine, Stavanger University Hospital, Norway; Department of Psychiatry and Neurochemistry, the Sahlgrenska Academy at the University of Gothenburg, Sweden; Department of Old Age Psychiatry, King's College London, UK	Drafting/revision of the manuscript for content, including medical writing for content; major role in the acquisition of data; additional contributions: administrative, technical, or material support
Dag Aarsland, MD, PhD	Centre for Age-Related Medicine, Stavanger University Hospital, Norway; Department of Old Age Psychiatry, King's College London, UK	Drafting/revision of the manuscript for content, including medical writing for content; major role in the acquisition of data; additional contributions: administrative, technical, or material support
Henrik Zetterberg, MD, PhD	Department of Psychiatry and Neurochemistry, the Sahlgrenska Academy at the University of Gothenburg; Clinical Neurochemistry Laboratory, Sahlgrenska University Hospital, Gothenburg, Sweden; Department of Neurodegenerative Disease, UCL Institute of Neurology, London; UK Dementia Research Institute at UCL, London; Hong Kong Center for Neurodegenerative Diseases, China; Wisconsin Alzheimer's Disease Research Center, University of Wisconsin School of Medicine and Public Health, Madison	Drafting/revision of the manuscript for content, including medical writing for content; major role in the acquisition of data
Alessandro Padovani, MD, PhD	Neurology Unit, Department of Clinical and Experimental Sciences, University of Brescia, Italy	Drafting/revision of the manuscript for content, including medical writing for content; major role in the acquisition of data
Laura Bonanni, MD, PhD	Department of Medicine and Aging Sciences, University G. d'Annunzio of Chieti-Pescara, Chieti, Italy	Drafting/revision of the manuscript for content, including medical writing for content; major role in the acquisition of data
Brit Mollenhauer	Department of Neurology, University Medical Center Göttingen; Paracelsus-Elena-Klinik Kassel, Kassel, Germany	Drafting/revision of the manuscript for content, including medical writing for content; major role in the acquisition of data
Sebastian Schade, MD	Paracelsus-Elena-Klinik, Kassel Germany	Drafting/revision of the manuscript for content, including medical writing for content; major role in the acquisition of data
Rik Vandenberghe, MD, PhD	Department of Neurosciences, KU Leuven, Belgium	Drafting/revision of the manuscript for content, including medical writing for content; major role in the acquisition of data
Koen Poesen, MD, PhD	Department of Neurosciences, KU Leuven, Belgium	Drafting/revision of the manuscript for content, including medical writing for content; major role in the acquisition of data
Milica G. Kramberger, MD, PhD	Department of Neurology and Medical Faculty, University Medical Center Ljubljana, Slovenia; Department of Neurobiology, Karolinska Institutet, Huddinge, Sweden	Drafting/revision of the manuscript for content, including medical writing for content; major role in the acquisition of data
Claire Paquet	Université de Paris Cité, Centre de Neurologie Cognitive, France	Drafting/revision of the manuscript for content, including medical writing for content; major role in the acquisition of data
Olivier Bousiges, PharmD, PhD	Laboratory of Biochemistry and Molecular Biology, University Hospital of Strasbourg; University of Strasbourg and CNRS, Strasbourg, France	Drafting/revision of the manuscript for content, including medical writing for content; major role in the acquisition of data
Benjamin Cretin	University of Strasbourg and CNRS; Memory Resource and Research Centre, University Hospital of Strasbourg, France	Drafting/revision of the manuscript for content, including medical writing for content; major role in the acquisition of data
Eline A.J. Willemse	Department of Laboratory Medicine, Amsterdam UMC, the Netherlands; Department of Neurology, Multiple Sclerosis Center and Research Center for Clinical Neuroimmunology and Neuroscience Basel; Departments of Biomedicine and Clinical Research, University Hospital Basel and University of Basel, Switzerland	Drafting/revision of the manuscript for content, including medical writing for content; major role in the acquisition of data; additional contributions: supervision
Charlotte E. Teunissen, PhD	Department of Laboratory Medicine, Amsterdam UMC, the Netherlands	Drafting/revision of the manuscript for content, including medical writing for content; major role in the acquisition of data; study concept or design; additional contributions: obtained funding; supervision
Afina W. Lemstra	Alzheimer Center Amsterdam, Amsterdam UMC, the Netherlands	Drafting/revision of the manuscript for content, including medical writing for content; major role in the acquisition of data; study concept or design; additional contributions: administrative, technical, or material support; supervision

## Appendix 2 Coinvestigators

**Table:**

Name	Location	Role	Contribution
Claudia Carrarini, MD	Department of Neuroscience, Imaging, and Clinical Sciences, G. d'Annunzio University of Chieti-Pescara, Italy	Site investigator	Data collection
Julien Dumurgier, MD, PhD	Centre de Neurologie Cognitive, GHU APHP Nord Hôpital Lariboisière Fernand-Widal, Paris, France	Site investigator	Data collection
Claire, Hourregue, MD	Centre de Neurologie Cognitive, GHU APHP Nord Hôpital Lariboisière Fernand-Widal, Paris, France	Site investigator	Data collection
Sinead Gaubert, MD	Centre de Neurologie Cognitive, GHU APHP Nord Hôpital Lariboisière Fernand-Widal, Paris, France	Site investigator	Data collection
Maximilien Porché, MD	Centre de Neurologie Cognitive, GHU APHP Nord Hôpital Lariboisière Fernand-Widal, Paris, France	Site investigator	Data collection
Matthieu Lilamand, MD, PhD	Centre de Neurologie Cognitive, GHU APHP Nord Hôpital Lariboisière Fernand-Widal, Paris, France	Site investigator	Data collection
Frédéric Blanc, MD, PhD	Memory Resource and Research Centre, Geriatrics Day Hospital, Geriatrics Department, University Hospital of Strasbourg; University of Strasbourg and CNRS, ICube Laboratory UMR 7357 and FMTS (Fédération de Médecine Translationnelle de Strasbourg), IMIS team, France	Principal investigator	Data collection
Pierre Anthony, MD	CM2R, Neuropsychology Unit, Head and Neck Department, Neurology Department, University of Strasbourg; CM2R, Geriatric Day Hospital, Geriatrics Division, Civil Hospitals of Colmar, France	Site investigator	Data collection
Catherine Demuynck, MD	Memory Resource and Research Centre, Geriatrics Day Hospital, Geriatrics Department, University Hospital of Strasbourg; University of Strasbourg and CNRS, ICube Laboratory UMR 7357 and FMTS (Fédération de Médecine Translationnelle de Strasbourg), IMIS team, France	Site investigator	Data collection
Catherine Martin-Hunyadi, MD	Centre Mémoire, de Ressources et de Recherche d'Alsace, Strasbourg-Colmar, France	Site investigator	Data collection
Candice Muller, MD	Memory Resource and Research Centre, Geriatrics Day Hospital, Geriatrics Department, University Hospital of Strasbourg; University of Strasbourg and CNRS, ICube Laboratory UMR 7357 and FMTS (Fédération de Médecine Translationnelle de Strasbourg), IMIS team, France	Site investigator	Data collection
Nathalie Philippi, MD	Memory Resource and Research Centre, Geriatrics Day Hospital, Geriatrics Department, University Hospital of Strasbourg; University of Strasbourg and CNRS, ICube Laboratory UMR 7357 and FMTS (Fédération de Médecine Translationnelle de Strasbourg), IMIS team, France	Site investigator	Data collection
Alix Ravier, MD	CM2R (Memory Resource and Research Centre), Geriatrics Department, University Hospitals of Strasbourg, France	Site investigator	Data collection
Anne Botzung, PhD	Memory Resource and Research Centre, Geriatrics Day Hospital, Geriatrics Department, University Hospital of Strasbourg; University of Strasbourg and CNRS, ICube Laboratory UMR 7357 and FMTS (Fédération de Médecine Translationnelle de Strasbourg), IMIS team, France	Site investigator	Data collection
Timothée Albasser	CM2R (Memory Resource and Research Centre), Day Hospital, Geriatrics Department, University Hospital of Strasbourg; 2 CNRS, ICube Laboratory, UMR 7357 and FMTS (Fédération de Médecine Translationnelle de Strasbourg), Team IMIS, University of Strasbourg, France	Site investigator	Data collection
Emmanuelle Epp-Ehrhardt	Memory Resource and Research Centre, Geriatrics Day Hospital, Geriatrics Department, University Hospital of Strasbourg, France	Site investigator	Data collection
Jeanne Merignac	Memory Resource and Research Centre, Geriatrics Day Hospital, Geriatrics Department, University Hospital of Strasbourg; University of Strasbourg and CNRS, ICube Laboratory UMR 7357 and FMTS (Fédération de Médecine Translationnelle de Strasbourg), IMIS team, France	Site investigator	Data collection
Laetitia Monjoin	Memory Resource and Research Centre, Geriatrics Day Hospital, Geriatrics Department, University Hospital of Strasbourg, France	Site investigator	Data collection
Isabelle Cleynen, PhD	Laboratory for Complex Genetics, Department of Human Genetics, KU Leuven, Belgium	Site investigator	Data collection
Mercè Boada, MD, PhD	Ace Alzheimer Center Barcelona, Institut Català de Neurociències Aplicades, Universitat Internacional de Catalunya-Barcelona, Centro de Investigación en Red-Enfermedades Neurodegenerativas (CIBERNED), Spain	Principal investigator	Data collection
Adela Orellana, PhD	Ace Alzheimer Center Barcelona, Institut Català de Neurociències Aplicades, Universitat Internacional de Catalunya-Barcelona, Centro de Investigación en Red-Enfermedades Neurodegenerativas (CIBERNED), Spain	Site investigator	Data collection

## Glossary

123I-FP-CIT: 123I-N-ω-fluoropropyl-2β-carbomethoxy-3β-(4-iodophenyl) nortropane
α-syn: α-synuclein
A+: amyloid abnormal
A−: amyloid normal
Aβ: β-amyloid
AD: Alzheimer disease
AIC: Akaike information criterion
DLB: dementia with Lewy bodies
E-DLB: European-Dementia With Lewy Bodies
GFAP: glial fibrillary acidic protein
HC: healthy control
MMSE: Mini-Mental State Examination
NfL: neurofilament light
PD: Parkinson disease
P-tau: phosphorylated-tau
RBD: REM sleep behavior disorder
